# Dipyridamole modulates the innate immune response during human endotoxemia

**DOI:** 10.1186/cc9686

**Published:** 2011-03-11

**Authors:** B Ramakers, NP Riksen, TH Stal, S Heemskerk, P Van den Broek, JG Van der Hoeven, P Smits, P Pickkers

**Affiliations:** 1 Radboud University Nijmegen Medical Centre, Nijmegen, the Netherlands

## Introduction

Previous studies have shown that the endogenous nucleoside adenosine is able to modulate inflammation and to prevent associated organ injury. Dipyridamole, an adenosine re-uptake inhibitor, increases extracellular adenosine concentrations during unfavorable conditions (for example, inflammation), and as such may modulate the inflammatory response. We examined the effects of dipyridamole treatment on innate immunity during human experimental endotoxemia.

## Methods

In a randomized double-blind placebo-controlled study, 20 healthy subjects received 2 ng/kg *Escherichia coli *endotoxin intravenously following 7-day pretreatment with dipyridamole, 200 mg retard twice daily, or placebo.

## Results

**Figure 1:**
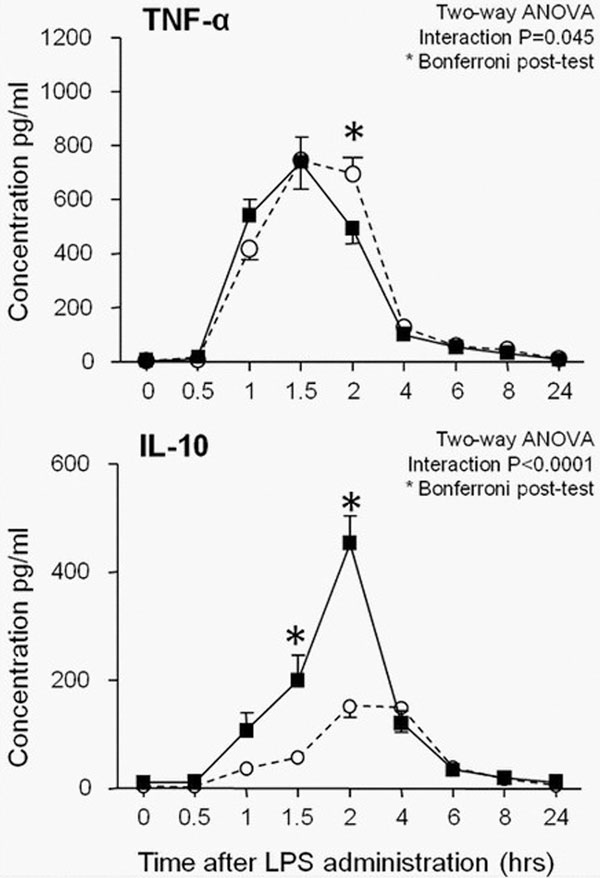
**Cytokine response following endotoxemia**.

Nucleoside transporter activity was significantly reduced by dipyridamole treatment with 89 ± 2% (*P *< 0.0001) and resulted in significantly augmented endogenous adenosine levels. Plasma concentrations of dipyridamole correlated with the peak adenosine concentration 2 hours after LPS administration (*r *= 0.82, *P *= 0.0038) and significantly augmented the anti-inflammatory IL-10 response during endotoxemia (*P *< 0.0001; Figure 1), an effect that correlated with the dipyridamole-induced increase in adenosine (*r *= 0.82; *P *= 0.0035). Finally, IL-10 peak concentrations were associated with a more pronounced decline in TNFα (*r *= 0.54, *P *= 0.018).

## Conclusions

Dipyridamole treatment increases adenosine concen-trations during systemic inflammation associated with an augmented anti-inflammatory response and a faster decline in TNFα during human experimental endotoxemia.

